# American Dental Society of Europe

**Published:** 1883-08

**Authors:** C. M. Wright


### CORRESPONDENCE.

### American Dental Society of Europe.

EDITOR OF REGISTER. As you and I cannot be present at the meeting of this wide-awake society this year, as we were a couple of years ago when the meeting was held in that charming watering place, Wiesbaden, suppose we sit down together and mourn over our loss. Let us go over the "programme," or bill of fare of the intellectual repast the society offers. Ah! This *is* a society! Born eleven years ago on the top of Rigi Mountain on a 4th of July, 6,000 feet above the sea, in full view of the whole range of Swiss lakes nestling in the valleys, and of the stately snow capped Alps—the poor little thing was so weak that its continued existence was a most uncertain problem. Now look at it! Does it keep up with the procession? Does it take the cake? Is it interested in the *science* of dentistry? Is it behind home societies in its regard for the *art* of dentistry? No, indeed! The men who are holding up the banner of "American dentistry" in the different sections of Europe, are called together but once a year to be sure, but they bring for exchange the *work* in the way of recorded observation, experiments, microscopic specimens and study of a year. They bring enthusiasm and love for their profession. They bring pure hearts filled with a love of truth and clean hands without a stain of professional jealousy, and "rusty axes." Oh, its a sturdy society. Look at Miller and his patient work in the study of obscure *causes* of the disease that supports the dental profession—the disease that caused our profession and that gives to us our daily bread. Look at Blount, as enthusiastic in the art, as the veriest boy in the profession. Look at Jenkins and his therapentic filling of tin and gold. Look at ourselves and see how much we here in America have learned and are influenced by the scientific work of these American dentists of Europe. We are proud of them and we have a right to be proud of them for the good reputations they give to our chosen profession—our American profession. God bless 'em, every one! As we can't go to Cologne let us saturate ourselves with the water of the old Rhine city, and as the odor permeates the air and is taken cognizance of by our brains may we think of our friends across the pond and wish them continued prosperity in their work in science's cause. C. M. WRIGHT.

The American Dental Society of Europe will hold its eleventh annual meeting at Cologne, commencing on Tuesday, August 7, 1883, at 10 o'clock. The meeting will be held in the Grand Hotel Victoria, where all members will please report on arrival.

PROGRAMME.

1. Experimental Tests of various Amalgams. Dr. W. St. George Elliott.
2. Experimental Tests of various Cements. Dr. E. A. Galbreath.
3. Pathology and Treatment of Rigg's Disease (Pyorrhoea Alveolaris). Dr. Geo. Mills (Brooklyn).
4. A Day's Practice. Dr. N. S. Jenkins.
5. Cause of the failure of Gold as a Filling Material. Dr. A. A. Blount.
6. Etiology of Dental Caries. Dr. W. D. Miller.
7. Dentistry of Electricity. Dr. John W. Crane.
8. Suggestions for a dental Notation, being a Code of Symbols for the Use of Dentists in recording Surgery Work. Dr. Geo. Cunningham.
9. Antiseptic Treatment of Diseases of the Dental Pulp. Dr. Rosenthal.

The following clinics will also be given during the session :

1. By Dr. N. S. Jenkins, demonstrating the use of tin and gold combined.
2. By Dr. A, A. Blount, demonstrating his method of lining cavities with noncohesive gold and completing with cohesive.
3. By Dr. W. St. George Elliott, demonstrating the use of the Electric Mallet with smooth points.
4. By Dr. E. DeTrey, demonstrating the operation for Resection and Cicatrization of the dental Pulp.

